# Core-Sheath Electrospun Nanofibers Based on Chitosan and Cyclodextrin Polymer for the Prolonged Release of Triclosan

**DOI:** 10.3390/polym14101955

**Published:** 2022-05-11

**Authors:** Safa Ouerghemmi, Stéphanie Degoutin, Mickael Maton, Nicolas Tabary, Frédéric Cazaux, Christel Neut, Nicolas Blanchemain, Bernard Martel

**Affiliations:** 1 Univ. Lille, CNRS, INRAE, Centrale Lille, UMR 8207-UMET-Unité Matériaux et Transformations, F-59000 Lille, France; safa.ouerghemmi@gmail.com (S.O.); nicolas.tabary@univ-lille.fr (N.T.); frederic.cazaux@univ-lille.fr (F.C.); bernard.martel@univ-lille.fr (B.M.); 2 Univ. Lille, Inserm, CHU Lille, U1008, Controlled Drug Delivery Systems and Biomaterials, F-59000 Lille, France; mickael.maton@univ-lille.fr (M.M.); nicolas.blanchemain@univ-lille.fr (N.B.); 3 Univ. Lille, Inserm, CHU Lille, U1286 INFINITE, Laboratory of Bacteriology, College of Pharmacy, F-59000 Lille, France; christel.neut@univ-lille.fr

**Keywords:** core-sheath nanofibers, chitosan, cyclodextrin polymer, polyelectrolyte complex, triclosan release, antimicrobial properties

## Abstract

This work focuses on the manufacture of core-sheath nanofibers (NFs) based on chitosan (CHT) as sheath and cyclodextrin polymer (PCD) as core and loaded with triclosan (TCL). In parallel, monolithic NFs consisting of blended CHT-PCD and TCL were prepared. Nanofibers were characterized by scanning electron microscopy (SEM), transmission electron microscopy (TEM), and Fourier Transform Infrared spectroscopy (FTIR). SEM displayed the morphology of NFs and the structure of the nanowebs, while TEM evidenced the core-sheath structure of NFs prepared by coaxial electrospinning. The core diameters and sheath thicknesses were found dependent on respective flow rates of both precursor solutions. Nanofibers stability and TCL release in aqueous medium were studied and correlated with the antibacterial activity against *Staphylococcus aureus* and *Escherichia coli*. Results showed that the release profiles of TCL and therefore the antibacterial activity were directly related to the type of nanofibers. In the case of monolithic nanofibers, the NFs matrix was composed of polyelectrolyte complex (PEC formed between CHT and PCD) and resulted in a prolonged release of TCL and a sustained antibacterial effect. In the case of core-sheath NFs, the PEC was formed only at the core-sheath interface, leading to less stable NFs and therefore to a faster release of TCL, and to a less extended antibacterial activity compared to monolithic ones.

## 1. Introduction

Over the past decade, nanofibrous electrospun materials have proven their interest in the biomedical field, especially for drug delivery purposes [1,2,3]. Indeed, electrospinning is a versatile process that allows the design of nanofibrous scaffolds based on a wide range of natural and/or synthetic polymers and exhibiting an interconnected porous structure with high surface area to volume ratio, compared to conventional nonwovens. These properties lead to a high capacity to either content drugs inside the fibers or adsorb drugs on the fibers through physical interactions or bonded by chemical interactions.

Among the natural polymers used for the design of nanofibrous drug delivery carriers, polysaccharides have been widely studied thanks to their biocompatibility and biodegradability [4,5,6,7]. Due to their hydrophilic functional groups, these polymers can stabilize macromolecular assemblies, such as polyelectrolyte complexes (PECs) for instance. PECs are formed in solution through the electrostatic interactions of oppositely charged polyelectrolytes. In particular, the electrospinning of chitosan (CHT) has been widely reported [8,9,10,11]. As a cationic polysaccharide, it possesses amine groups which are protonated at low pH and is therefore soluble in acidic conditions. In solution, chitosan easily forms polyelectrolyte complexes with polyanions [12]. Recently, we reported the PECs formation between chitosan and a cyclodextrin-citric acid crosslinked anionic polymer (PCD) developed in our group, for the build-up of drug delivery systems in the shape of multilayer coatings on textile [13,14] and metallic substrates [15], hydrogels [16], and sponges [17,18] for wound dressing, cardiovascular applications, and bone regeneration. CDs and their polymers present hydrophobic cavities allowing the formation of reversible inclusion complexes with various active principles, improving their solubility and bioavailability [19]. Some studies report the electrospinning of cyclodextrins in their pristine form blended with other polymers [20,21,22,23] or cyclodextrins in a polymerized form [24]. In previous papers, we studied the electrospinning of CHT-PCD blends for the production of monolithic nanofibers (NFs) loaded with triclosan (TCL) [25] and simvastatin [26] for wound dressing and cardiovascular applications, respectively. Results showed that the PEC based on CHT and PCD improved the stability of the monolithic NFs in physiologic pH and acidic conditions and reduced the diffusion of the PCD/active molecules inclusion complexes from the NFs. Moreover, the inclusion complexes led to a sustained release of TCL and simvastatin in dynamic conditions, resulting in the prolonged antibacterial effect against Gram-positive and Gram-negative bacteria in the case of TCL.

Drug release from NFs systems present profiles that depend on extrinsic (pH, temperature, etc.) and intrinsic parameters, with the latter concerning their strategy of incorporation in the nanofibrous mats (on the surface or in the NFs matrix), the hydrophobicity of the drug and its crystalline or amorphous state, the type of interactions of the active principle with the NFs surface, the nature of the polymer matrix, the swelling of NFs, and also the nanofibers structure and morphology. Indeed, the electrospinning of a polymer/drug mixture offers the possibility to vary the method of drug embedding within the nanofibers [1,27]. The post-immobilization of pharmaceutical active principles on NFs can be achieved through chemical (covalent) or physical (van der Waals, electrostatic, hydrophobic, or hydrogen) interactions. In the case of covalent immobilization, the drug release is correlated with the polymer degradation rate or with the reversibility of the covalent bonding (ester, imine) [28]. In the case of physical interactions, the drug release is more rapid and depends on the release conditions (presence of salt in the release medium) [1]. In the case of bulk incorporation of the drug, the drug release can be controlled by a diffusion of the drug through the matrix and by the degradation of the matrix (for biodegradable polymers) [27]. Another method to monitor the drug release rate and profile is to design multilayered systems by depositing successively electrospun layers of different polymers [29]. With this method, multidrug release could be achieved, and the release profiles depend on drug/polymer interaction and on the number of layers. An alternative approach consists in electrospinning a solution containing drug encapsulated nanoparticles, which may be either polymer or inorganic nanoparticles [30]. The active principle release would in particular depend on the degradation/swelling properties of the nanoparticles and nanofiber matrices. Finally, the electrospinning technique can be used to design core-sheath nanofibers through emulsion or coaxial electrospinning [31]. With emulsion electrospinning, the solution contains a water-soluble polymer, a non-water-soluble polymer, and an emulsifier to stabilize both phases. The drug is generally mixed in the less concentrated phase which agglomerates in the dispersed drop and finally forms the core of the obtained nanofibers. Coaxial electrospinning requires the use of a specific coaxial nozzle system in which two different solutions are simultaneously electrospun [32]. In most cases in coaxial electrospinning, the drug is incorporated in the core solution to achieve a reservoir effect and target a prolonged release [33,34]. As for the electrospinning of a single polymer, coaxial process is affected by environmental conditions, solution and process parameters, but with additional complications: solvent miscibility, electrical conductivity, and flow rates of the two solutions. In the literature, the coaxial electrospinning of chitosan was reported [8,35,36]. Chitosan was for instance associated with synthetic biodegradable polymers such as polycaprolactone [37] and polylactic acid [38] or with peptidic polymers such as gelatin [39]. However, the electrospinning of chitosan with polymeric anions is less investigated in the literature, as this association is a challenging one that requires a mastery of the electrospinning process and parameters that lead to a continuous electrospinning without gel formation at the tip of the nozzle resulting from PEC formation. Nista et al. published the coaxial electrospinning of alginate (core) and chitosan (sheath) where they added a third high molecular weight polymer in both solutions, such as poly(ethylene oxide) (PEO), to obtain a feasible process [40]. Concerning core-sheath nanofibers based on CDs, some recent studies were published. Inclusion complexes of drug and hydroxypropyl-β-cyclodextrin (HPβCD) have been incorporated in the core structure and presented a prolonged release of the studied active principles [41,42]. Moreover, Kaszoki et al. reported the electrospinning of inclusion complexes of two different drugs with HPβCD combined with polylactic acid or polyvinylpyrrolidone in both core and sheath layers, respectively, and focused on the investigation of the morphological and chemical structure by SEM, TEM, Raman, and X-Ray photoelectron spectroscopy (XPS) [43]. In another study, core-sheath nanofibers based on polyvinylbutyral (PVB) as core and PVB/β-CD/hexamethylene diisocyanate as sheath were stabilized by a post-thermal treatment leading to the crosslinking of the sheath layer [44]. Gao et al. functionalized poly(vinylidene fluoride)-polystyrene core-sheath nanofibers by successive post-immersions in dopamine and β-CD solutions and studied their adsorption capacity towards organic pollutants through the formation of inclusion complexes [45]. β-CD grafted onto graphene oxide as core solution and chitosan as sheath solution were also electrospun to develop co-delivery systems targeting the release of anticancer and antimicrobial drugs [35]. Coaxial electrospinning was also used to design NFs based on drug loaded cyclodextrin polymer (prepared by crosslinking β-CD with epichlorhydrin) as core and polymethylmethacrylate as sheath [24]. A thermal treatment was applied to induce the crosslinking between both polymers and increase drug retention. Finally, another approach consisted in the use of pseudopolyrotaxanes of star-polycaprolactone (star-PCL) with β-CD as sheath solution electrospun with PCL as core solution to obtain nanofibers with enhanced surface reactivity [46].

The goal of this study was to develop core-sheath nanofibers with anionic cyclodextrin polymer complexing triclosan as core and chitosan as sheath by coaxial electrospinning. The effect of inner and outer solutions flow rates on the nanofibrous structure and size was investigated by SEM and TEM to assess the obtained morphologies. Core-sheath nanofibers obtained from coaxial needle were compared to classical monolithic ones, in terms of stability and drug release in aqueous medium at pH 7.4 and antibacterial effect against *Staphylococcus aureus* and *Escherichia coli* evidenced by Kirby Bauer method. Results are discussed accordingly with the cyclodextrin polymer and chitosan distribution in the nanofibers, i.e., blended (bulk PEC) or forming core and sheath (PEC at the core-sheath interface).

## 2. Materials and Methods

### 2.1. Materials

Chitosan (CHT, low molecular weight grade, M_v_ 95,000 g·mol^−1^, 94.3% deacetylated, Sigma Aldrich, Saint-Quentin Fallavier, France, batch number MKBL7900 V), Polyethylene oxide (PEO, 900,000 g·mol^−1^), glacial acetic acid (AA), citric acid (CTR), sodium hypophosphite monohydrate (NaH_2_PO_2_·H_2_O), phosphate buffered saline (PBS), and triclosan (5-chloro-2-(2,4-dichlorophenoxy) phenol, TCL) were purchased from Sigma Aldrich (Saint-Quentin Fallavier, France). (2-hydroxypropyl)-β-cyclodextrin (HPβCD) Kleptose^®^ HP MS = 0.62 was provided by Roquette (Lestrem, France).

All reagents were used as received from the manufacturer without further purification. Ultrapure water was used for all experiments (Veolia water aquadem, Purelab flex, ELGA, 18.2 MΩ).

### 2.2. Synthesis and Characterization of Cyclodextrin Polymer

The anionic polymer (PCD) of HPβCD crosslinked by CTR was synthesized according to a method previously described by Martel et al. [19,25]. According to characterization analyses already published [25], its weight content in HPβCD moieties was 50% (determined by ^1^H NMR) and its average molecular masses in weight and number (Mw and Mn) were 64,650 g·mol^−1^ and 13,500 g·mol^−1^, respectively (measured by size exclusion chromatography in water). The number of residual carboxylic acid groups was estimated to 4.00 ± 0.10 mmol of COOH functions per gram of PCD (determined by acid-base titration method).

### 2.3. Electrospinning

Different polymer compositions were prepared in order to produce monolithic and core-sheath nanofibers. PEO was systematically added in electrospun solutions in order to enhance the chain entanglements and hydrogen bonds to promote the formation of a continuous jet and nanofibers deposition on the collector [47].

**Figure 1:**
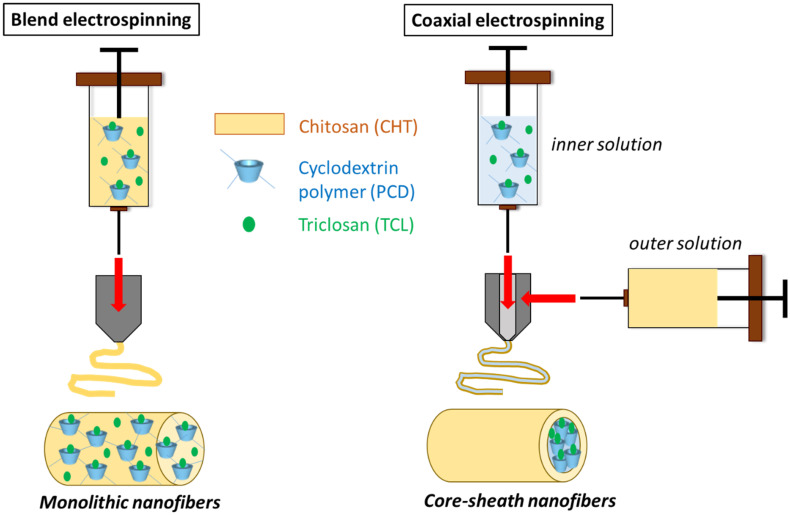
Schematic presentation of the preparation of monolithic and core-sheath nanofibers based on chitosan, cyclodextrin polymer, and triclosan.

Monolithic nanofibers (M0 and M1) were generated from classical needle (Figure 1). PolyHPβCD (PCD) with a concentration of 8% *w/v* was first dissolved in water. A total of 5 wt% of TCL according to total polymers concentrations (CHT, PCD, and PEO) was added to PCD solution. The mixture was kept under stirring for 72 h at 37 °C. CHT and PEO powders mixture (9:1) (*w/w*) was then added to PCD/TCL solution. Finally, glacial acetic acid was added in order to obtain a final concentration of acid of 90% (*v/v*), and the clear solution was stirred overnight at room temperature. As control to evidence the role of PCD on the release and antibacterial effect of TCL, a solution based on a mixture of CHT, PEO, and 5 wt% of TCL was also prepared. The obtained solutions were then loaded into a 5 mL plastic syringe connected to a 21-gauge needle (Terumo Europe, Leuven, Belgique) by the intermediate of a polyethylene catheter (inner diameter 1 mm, Vygon, Ecouen, France) and placed onto a syringe pump (Fisher Scientific, Illkich, France). A high voltage was then applied, and the nanofibers were collected on a roll collector (diameter 80 mm, speed 100 rpm). The optimal electrospinning parameters were determined in a previous study as follows: a flow rate of 0.5 mL/h, an applied voltage of 13 kV, and a tip-to-collector distance of 200 mm [25]. Relative humidity and temperature were fixed at 35 ± 2% and 20 ± 2 °C, respectively. Finally, the nanofibers were submitted to a heat treatment at 90 °C in order to improve their stability in aqueous medium and stored in desiccators containing silica gel at room temperature.

Core-sheath nanofibers (CS1 to CS4) were prepared by coaxial electrospinning (Figure 1). The inner solution was prepared as follows: the inclusion complex of PCD (8% *w/v*)/TCL (5 wt% according to total core + sheath polymer concentrations) and PEO (2 wt% (*w/v*)) were dissolved in glacial acetic acid/water at a concentration of 50% (*v/v*). The outer solution was prepared by dissolving a mixture of 3.5 wt% of CHT and PEO (9:1) in glacial acetic acid/water at a concentration of 90% (*v/v*). The electrospinning apparatus was equipped with a coaxial system (Linari Nanotech, Pisa, Italy, inner needle 21 gauge, outer needle 15 gauge). The two solutions were loaded into two separate 5 mL syringes placed onto syringe pumps (Fisher Scientific, Illkirch, France) connected to the coaxial system through polyethylene catheters (inner diameter 1 mm, Vygon, Ecouen, France). The tip-to-collector distance was set to 200 mm and the applied voltage was adjusted to 15–20 kV. The flow rate was varied for both inner and outer solutions in order to study its effect on core-sheath structure. Relative humidity and temperature were fixed at 35% ± 2% and 20 ± 2 °C, respectively. Finally, the nanofibers were submitted to a heat treatment at 90 °C in order to improve their stability in aqueous medium and stored in desiccators containing silica gel at room temperature.

The solution compositions and experimental conditions are given in Table 1 with the nomenclature of prepared nanofibrous samples. The theoretical proportions of each component in core-sheath nanofibers were calculated according to the applied flow rates. The inclusion complex formation between PCD and TCL has been characterized in a previous paper [25].

### 2.4. Nanofibers Characterization

The morphology and diameter size of the nanofibers were analyzed using SEM (Hitachi S-4700 SEM field emission GU) operating at an accelerating voltage of 5 kV and an emission current of 10 µA. Samples were sputtered beforehand with a thin layer of chrome. Diameter sizes were calculated using the software ImageJ as an average of 50 nanofibers diameter measurements.

The core-sheath structure was investigated using TEM (Tecnai FEI TEM G2-20 twin equipped with a LaB6 filament) operating at an accelerating voltage of 200 kV.

The chemical structure of nanofibers was studied by Attenuated Total Reflectance—Fourier Transformed InfraRed (ATR-FTIR) spectroscopy using a PerkinElmer spectrometer (Spectrum One) equipped with Spectrum software. Spectra were recorded from 4000 cm^−1^ to 650 cm^−1^ (scan number = 8) with a resolution of 4 cm^−1^.

### 2.5. Nanofibers Stability in PBS

Nanofibrous samples weighing approximately 20 mg were cut and placed in PBS at pH 7.4. At different time intervals, the sample was removed, rinsed with distilled water, dried at 37 °C, and finally weighed with a precision balance (KERN, ALJ 220—5DNKM). Measures were conducted in triplicates.

The weight loss of the membranes (WL) was calculated according to the following Equation (1):

WL (%) = (W_0_ − W_t_) × 100/W_0_ (1)

where W_0_ = initial dry weight and W_t_ = dry weight at specific time (t).

### 2.6. TCL Release

The release of TCL in PBS at pH 7.4 was carried out in batch. A total of 20 mg of nanofibrous membranes loaded with TCL were placed into 150 mL of PBS under agitation (100 rpm) in a thermostatically controlled oscillating oven at 37 °C (Thermoshake, Gerhardt, Les Essarts-le-Roi, France). At each predefined time, aliquots (3 mL) of the soaking solution were transferred into quartz cells (1 cm). The absorbance was measured at a wavelength of 282 nm using a UV spectrometer (Shimadzu UV-1800). Withdrawn aliquots were then returned into the batch solutions in order to keep the volume of the supernatant unchanged. Analyses were carried out in triplicates.

The release mechanism was studied according to the mathematic model of Korsmeyer-Peppas [48] applied to the release kinetics profiles. This model describing the drug release from a polymer system is based on the following Equation (2):

M_t_/M∞ = kt^n^ (2)

where M_t_/M∞ is the percentage of accumulated drug for a specific time (t), n is the release exponent, and k is the release constant. This equation is only applicable for low release times and the part of the release profile where M_t_/M∞ is inferior to 0.6. This model classifies the release kinetics mechanisms depending on the value of the exponent n. In the case of cylindrical matrices, if 0.45 ≤ n, the release mechanism is considered as Fickian (drug diffusion); if 0.45 < n < 0.89 or n = 0.89 or n > 0.89, the mechanism is considered as non Fickian, which indicates that drug diffusion is combined with another mass transfer mechanism (polymer swelling, erosion).

### 2.7. Antibacterial Activity

The antibacterial activity of released medium from TCL loaded nanofibers was evaluated by the diffusion Kirby-Bauer test after sterilization by UV irradiation during 1 h for each face. TCL loaded nanofibers (Ø15 mm) were immersed in 5 mL of sterile PBS at 37 °C under agitation (80 rpm) with 100% renewal of the medium at different time intervals: 15 min, 30 min, then every hour up to 6 h, and finally every day up to 9 days. *S. aureus* (strain CIP 224) and *E. coli* (strain K12) were suspended in Ringer’s cysteinated diluent in order to obtain a stock suspension with a density of 1.0 × 10^4^ CFU/mL. A total of 18 mL of Mueller-Hinton agar (MHA) were poured in Petri dishes (Ø9 cm) and then, 0.1 mL of the *S. aureus* or *E. coli* stock suspension were thereafter seeded on the agar. Three holes of 6 mm in diameter were punched per Petri dish and 50 µL of the release medium were added into the holes. After 24 h incubation at 37 °C, the diameter of the inhibition zone was measured and plotted as a function of contact time in PBS. Inhibition tests were performed in triplicate for each time point.

## 3. Results and Discussion

### 3.1. Morphology Study of the Electrospun Nanofibers

**Figure 2:**
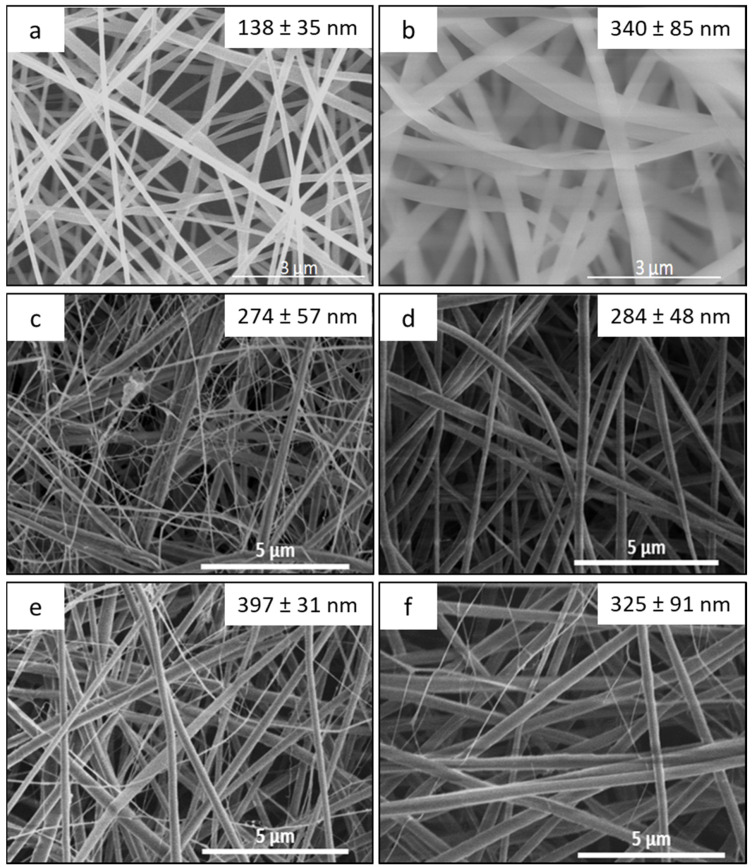
SEM pictures of monolithic (**a**) M0, (**b**) M1 and core-sheath (**c**) CS1, (**d**) CS2, (**e**) CS3, (**f**) CS4 nanofibers.

SEM pictures of monolithic and core-sheath NFs are shown in Figure 2. Average diameters are given in Table 1. In the case of monolithic nanofibers (Figure 2a,b), a smooth surface morphology was observed with or without PCD. The presence of PCD in M1 nanofibers led to an increase of the diameter of the nanofibers from 138 to 340 nm compared to M2, due to an increase of the viscosity of the electrospun solution [25].

For core-sheath nanofibers (Figure 2c–f), acetic acid concentrations of inner and outer electrospun solutions (50% (*v/v*) for core solution, 90% (*v/v*) for sheath solution) were chosen to prevent the formation of a polyelectrolyte complex between cationic CHT and anionic PCD at the needle output, which would induce the formation of aggregates and therefore would hinder the jet stretching. As a matter of fact, in concentrated organic acids, it can be considered that the carboxylic groups of PCD are protonated and consequently do not interact with CHT ammonium groups. Indeed, defect-free nanofibers were obtained by coaxial electrospinning for each flow rates settings. Average diameters of core-sheath nanofibers are also given in Table 2 and show that the diameter increases with the increase of inner and/or outer solution flow rates. This observation reveals that the effect of the flow rate in coaxial electrospinning is similar to the one in electrospinning of a monophasic solution [49]. Furthermore, the theoretical composition of core-sheath nanofibers was calculated considering the inner and outer solutions flow rates and is reported in Table 2. By increasing the flow rate of outer solution based on CHT/PEO, the PCD/TCL ratio in the nanofibers decreases.

SEM micrographs show that two classes of diameter ranges are obtained. For instance, in CS1 (Figure 2c), nanofibers can be divided into two groups: a primary group with an average diameter of 274 ± 57 nm and a secondary network with thinner nanofibers (<10 nm). This may be due to the division of the jet into several ones during the electrospinning. Electrostatic forces lead to the stretching of a core-sheath jet which solidifies by solvent evaporation before solid nanofibers deposition on the collector, and in the meantime, the outer solution is itself subject to electrostatic forces that eject secondary thinner jets based on CHT and PEO only. More homogeneous diameter ranges are obtained by increasing the gap between both flow rates (CS2 and CS3). However, by increasing inner and outer flow rates (CS4), the average diameter decreases and the dispersity increases. This may be explained by the increase of the voltage up to 20 kV, leading to an increase of electrostatic charges at the surface and therefore a greater stretching of the jet to produce thinner nanofibers [32].

**Figure 3:**
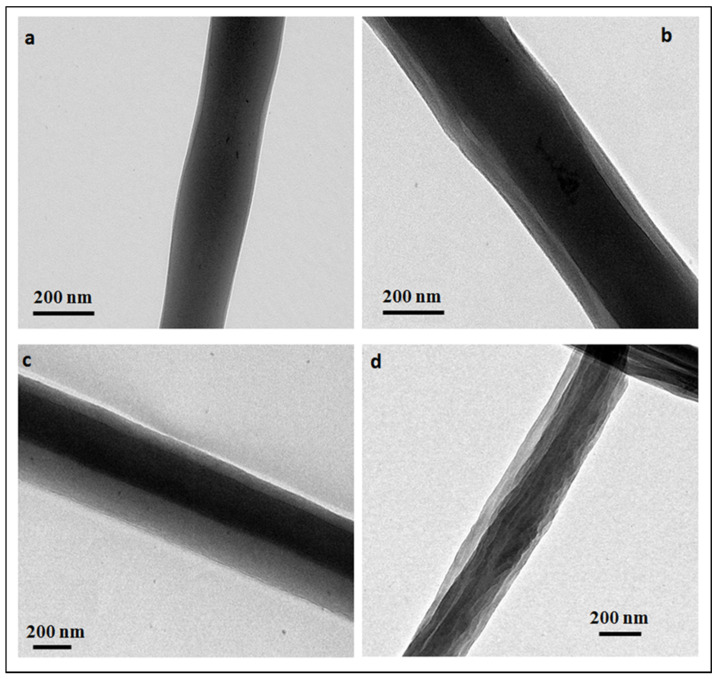
TEM images of core-sheath nanofibers (**a**) CS1, (**b**) CS2, (**c**) CS3, (**d**) CS4.

In order to evidence the core-sheath structure of nanofibers produced by coaxial electrospinning, samples were observed by TEM and micrographs are shown on Figure 3. The contrast observed on NFs due to the difference of composition of the polymers confirm the formation of core-sheath structures. However, some fibers present imperfect concentricity with a core not perfectly centered. Average core diameters and sheath thicknesses are given in Table 2.

For constant inner solution flow (samples CS1 to CS3), the sheath thickness increases with the outer solution flow. Indeed, by increasing the outer solution flow, a higher amount of CHT would wrap PCD/TCL leading to a higher sheath thickness. For constant outer solution flow (samples CS2 and CS4), the core diameter increases with the inner solution flow while the sheath thickness remains constant.

Moreover, FTIR-ATR spectra of core-sheath nanofibers (Supplementary Data Figure S1) present similar peaks to monolithic nanofibers ones [25]. The penetration depth of 0.5 mm of the FTIR beam does not allow to differentiate monolithic and core-sheath NFs.

### 3.2. Study of Stability in Aqueous Medium

**Figure 4:**
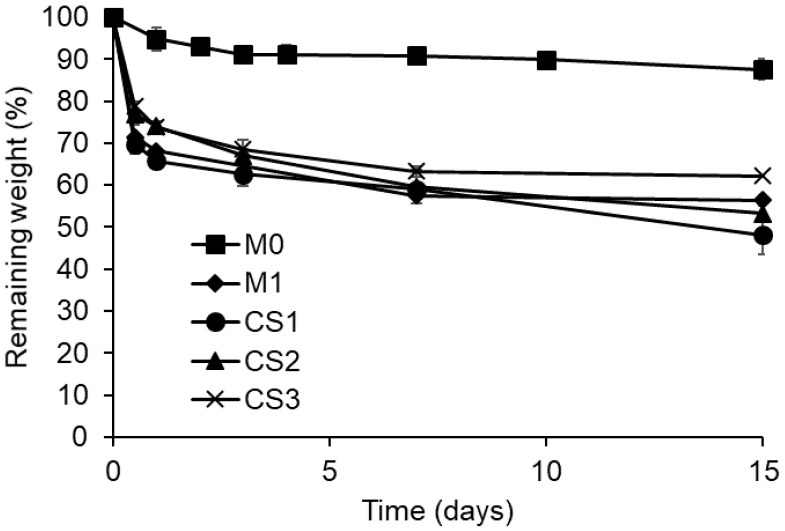
Weight loss at pH 7.4 at 37 °C within 15 days of degradation of monolithic and core-sheath nanofibers.

The weight loss of both monolithic and core-sheath NFs within time in PBS buffer at pH 7.4 is presented in Figure 4 and is almost stable. Monolithic NFs without PCD (M0) present a weight loss of around 10% after one day corresponding to PEO release [25]. NFs based on PCD exhibit a higher weight loss within 1 day of around 30%, 34%, 26%, and 27% for M1, CS1, CS2, and CS3, respectively, due to the dissolution of PEO and a part of PCD [25]. Despite these similar values, the relative proportions of dissolved PCD are higher in core-sheath nanofibers, considering their lower PCD and PEO contents compared to monolithic nanofibers as presented in Table 1. This higher weight loss may also be correlated to the thickness of core-sheath structure which would allow a faster diffusion of PCD and PEO. Indeed, the sample CS1 has the highest degradation rate and the lowest sheath thickness. Moreover, the fast degradation of thin nanofibers (<10 nm) observed on the SEM image (Figure 2c) may also contribute to the weight loss.

### 3.3. Triclosan Release

**Figure 5:**
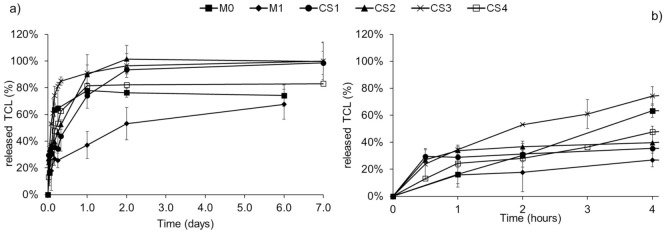
Triclosan release profiles in PBS at pH 7.4 at 37 °C from monolithic and core-sheath nanofibers (**a**) in a time period of 7 days and (**b**) within the first 4 h.

The release profiles of TCL from both monolithic and core-sheath NFs in PBS are compared in Figure 5. On one hand, within the first 4 h of release, an important burst effect is observed for CS3 and CS4 with a release of 74% and 58% of TCL after 4 h, respectively. A similar release profile to the one of monolithic nanofibers without cyclodextrin polymer (M0) is observed. In contrast, a reduced burst release is observed for CS1 and CS2 with a release of only 35% and 40% of TCL after 4 h, respectively. As a whole, results indicate that for core-sheath NFs, there is no obvious direct correlation between the release and the sheath thickness, probably due to the inhomogeneous thickness observed by TEM. Moreover, considering the loss of PEO and PCD (Figure 4) that simultaneously causes TCL release, results show that drug release kinetics was not controllable by the core and sheath ratio in the nanofibers.

On the other hand, the monolithic nanofibers based on cyclodextrin polymer (M1) exhibit a lower burst effect and a prolonged release up to 6 days. In monolithic nanofibers, interactions between CHT and PCD are dense. This is due to their intimate blending and to the formation of a polyelectrolyte complex leading to the homogeneous matrix of nanofibers, whereas this polyelectrolyte complex is formed only at the core-sheath interface. Therefore, core-sheath nanofibers are less water-resistant than monolithic ones and display a faster release of TCL, as revealed from degradation study and release tests.

After that burst effect, the drug is gradually released until a plateau raised after 7 days at 93%, 100%, 96.5%, and 90% for CS1, CS2, CS3, and CS4, respectively, and after 6 days at 68% for M1, confirming the role of the polyelectrolyte complex to retain the drug within the polymer network.

In order to determine the release mechanisms of TCL from the electrospun nanofibers, the Korsmeyer-Peppas model has been applied to the release kinetics and the results are shown in Table 3.

The correlation ratio R^2^ values confirm the good adequacy of the model with the experimental data. In all cases, except for CS4 nanofibers, the *n* value is lower than 0.45, indicating that the TCL release mechanism is governed by Fick’s law of diffusion. In the case of CS4 nanofibers, the *n* value higher than 0.45 implies that diffusion is not the only mechanism, but that swelling and/or degradation also play a role in the release of TCL.

### 3.4. Antibacterial Activity

**Figure 6:**
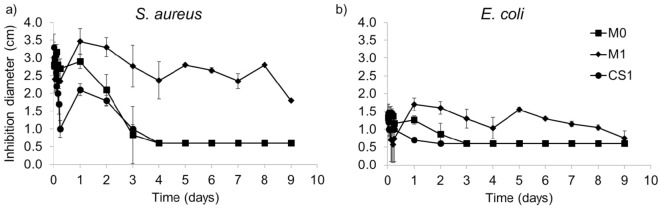
Antibacterial activity against (**a**) *S. aureus* and (**b**) *E. coli* of the released medium in PBS from TCL loaded monolithic and core-sheath nanofibers.

The antibacterial activity of the released medium from TCL loaded monolithic (M0 and M1) and core-sheath (CS1) nanofibers against *S. aureus* and *E. coli* was assessed to compare the two types of nanofibers and is presented in Figure 6. CS1 was chosen as the core-sheath sample with the lowest burst effect (Figure 5). As already published, the control samples without TCL did not exhibit any inhibition diameter and therefore display no antibacterial activity [25].

Figure 6 displays that the antibacterial activity of monolithic nanofibers is important during the first 24 h. It is worth noting that the concentration of released TCL was sufficient to observe an inhibition diameter either for *S. aureus* or *E. coli* for monolithic nanofibers, with a higher efficacy towards *S. aureus*. The antibacterial activity of monolithic nanofibers without PCD (M0) is observed up to 4 days for *S. aureus* and 3 days for *E. coli*. Monolithic nanofibers with PCD (M1) exhibit a significant antibacterial effect up to 9 days, correlated with the prolonged release of TCL within this period (Figure 5a).

In contrast, core-sheath nanofibers present a reduced antibacterial effect in terms of intensity and time compared to monolithic M1 nanofibers. The initial content of TCL is different in the two series (Table 1). Indeed, M1 nanofibers contain 5 wt% of TCL, whereas CS1 nanofibers contain 2.7 wt% of TCL. Moreover, the latter showed a faster release of TCL than monolithic nanofibers as well as a greater weight loss. These combined points would then lead faster to a concentration of TCL in the release medium lower than its minimal inhibitory concentration (MIC).

## 4. Conclusions

This study aimed at evaluating the potential of core-sheath nanofibers based on chitosan and cyclodextrin polymer for the release of an active principle. Characterization techniques confirmed the formation of the core-sheath structure with chitosan as sheath and cyclodextrin polymer as core, which sizes and degradation rates were controlled by tuning the electrospinning solution flow rates. The drug release and antibacterial assays consolidated the potential of the cyclodextrin polymer for the design of prolonged drug delivery nanofibrous systems. The comparison of monolithic and core-sheath nanofibers highlighted the improvement of anionic cyclodextrin polymer properties through the formation of a strong polyelectrolyte complex with cationic chitosan, especially when it is formed within the whole nanofibers instead of at the interface of both polymers. However, the choice of a core-sheath structure could be considered in the case of the incorporation of two non-miscible drugs, which would be compatible with the core and sheath solutions, respectively.

## Figures and Tables

**Table 1:** Composition of the electrospun solutions and applied flow rates and voltages (on the left part); theoretical weight compositions of the loaded nanofibers and observed diameters (on the right part).

**Nomenclature**	**Composition**		**Composition of** **Electrospun Solutions**				**Flow Rate mL/h**		**Voltage** **kV**	**Theoretical Composition of Nanofibers**				**Diameter** **nm**
			CHT % *w/v*	PEO % *w/v*	PCD % *w/v*	TCL * wt%				CHT wt%	PEO wt%	PCD wt%	TCL wt%	
M0	CHT/TCL		3.15	0.35	-	5	0.5		13	85.5	9.5	-	5	138 ± 35
M1	CHT + PCD8/TCL		3.15	0.35	8	5				40	4.4	50.6	5	340 ± 85
	**Sheath**	**Core**	**Outer Solution**		**Inner Solution**		**Outer**	**Inner**						**Total**
CS1	CHT	PCD/TCL	CHT/PEO 3.5% *w/v*		PCD 8% *w/v* PEO 2% *w/v* TCL * 5 wt%		0.4	0.3	15	51.4	13.7	32.2	2.7	274 ± 57
CS2	CHT	PCD/TCL					0.7	0.3	15	63.0	12.6	22.5	1.9	284 ± 48
CS3	CHT	PCD/TCL					0.9	0.3	15	67.5	12.2	18.7	1.6	397 ± 31
CS4	CHT	PCD/TCL					0.7	0.5	20	52.5	13.6	31.4	2.5	325 ± 91

* TCL is expressed from the weight ratio of TCL vs. CHT, PEO, PCD components of the NF matrix.

**Table 2:** Values of core diameters and sheath thicknesses of core-sheath nanofibers with corresponding electrospinning flow rates.

Sample	Inner Solution Flow mL/h	Outer Solution Flow mL/h	Core Average Diameter * nm	Sheath Average Thickness * nm	Nanofibers Average Diameter ** nm
CS1	0.3	0.4	198	14	274 ± 57
CS2	0.3	0.7	194	47	284 ± 48
CS3	0.3	0.9	157	87	397 ± 31
CS4	0.5	0.7	242	48	325 ± 91

* measured from TEM images, ** measured from SEM images.

**Table 3:** Correlation of the triclosan release kinetics of electrospun nanofibers with Korsmeyer-Peppas model and corresponding parameters.

Sample	R^2^	*n*	k	Mechanism
M0	0.80	0.28	8.5	Fickian
M1	0.90	0.19	6.0	Fickian
CS1	0.77	0.09	0.4	Fickian
CS2	0.95	0.21	0.6	Fickian
CS3	0.92	0.35	1.4	Fickian
CS4	0.97	0.54	1.1	Non Fickian

## Data Availability

All data produced in this study are presented in this paper.

## Supplementary Materials

The following supporting information can be downloaded at: https://www.mdpi.com/article/10.3390/polym14101955/s1, Figure S1: ATR-FTIR spectra of PEO, CHT, PCD powders and M1 and CS1 NFs.
