# Supplementary material for: Core-Sheath Electrospun Nanofibers Based on Chitosan and Cyclodextrin Polymer for the Prolonged Release of Triclosan
Source: Polymers (Basel). 2022 May 11;14(10):1955. doi: 10.3390/polym14101955 (PMC9147127; doi:10.3390/polym14101955)
Supplement: Supplementary file 1 [file polymers-14-01955-s001.zip › polymers-1665001-supplementary.pdf]

polymers

MDPI

Article

Core-Sheath Electrospun Nanofibers Based on Chitosan and Cyclodextrin Polymer for the Prolonged Release of Triclosan

Safa Ouerghemmi [1], Stéphanie Degoutin [1,*], Mickael Maton [2], Nicolas Tabary [1], Frédéric Cazaux [1], Christel Neut [3], Nicolas Blanchemain [2] and Bernard Martel [1]

[1] Univ. Lille, CNRS, INRAE, Centrale Lille, UMR 8207-UMET-Unité Matériaux et Transformations, F-59000 Lille, France; safa.ouerghemmi@gmail.com (S.O.); nicolas.tabary@univ-lille.fr (N.T.); frederic.cazaux@univ-lille.fr (F.C.); bernard.martel@univ-lille.fr (B.M.)
[2] Univ. Lille, Inserm, CHU Lille, U1008, Controlled Drug Delivery Systems and Biomaterials, F-59000 Lille, France; mickael.maton@univ-lille.fr (M.M.); nicolas.blanchemain@univ-lille.fr (N.B.)
[3] Univ. Lille, Inserm, CHU Lille, U1286 INFINITE, Laboratory of Bacteriology, College of Pharmacy, F-59000 Lille, France; christel.neut@univ-lille.fr
* Correspondence: stephanie.degoutin@univ-lille.fr

Citation: Ouerghemmi, S.; Degoutin, S.; Maton, M.; Tabary, N.; Cazaux, F.; Neut, C.; Blanchemain, N.; Martel, B. Core-Sheath Electrospun Nanofibers Based on Chitosan and Cyclodextrin Polymer for the Prolonged Release of Triclosan. *Polymers* **2022**, *14*, 1955. https://doi.org/10.3390/polym14101955

Academic Editor: Andreea-Teodora Iacob

Received: 18 March 2022
Accepted: 7 May 2022
Published: 11 May 2022

Publisher's Note: MDPI stays neutral with regard to jurisdictional claims in published maps and institutional affiliations.

Copyright: © 2022 by the authors. Licensee MDPI, Basel, Switzerland. This article is an open access article distributed under the terms and conditions of the Creative Commons Attribution (CC BY) license (https://creativecommons.org/licenses/by/4.0/).

Supplementary Information

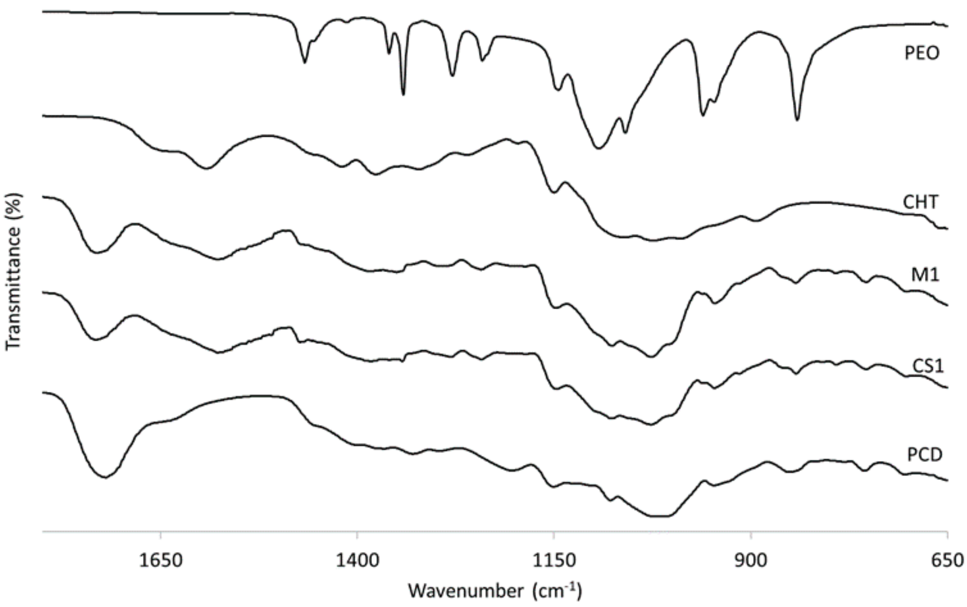

Figure S1. ATR-FTIR spectra of PEO, CHT, PCD powders and M1 and CS1 NFs.
